# “God Didn’t Make a Mistake in Creating Me”: Intrapersonal Resilience Processes among Gay and Bisexual Male Youth in Kenya

**DOI:** 10.3390/adolescents1030020

**Published:** 2021-07-13

**Authors:** Gary W. Harper, Katherine A. Lewis, Gabriella A. Norwitz, Elijah Ochieng Odhiambo, Laura Jadwin-Cakmak, Felix Okutah, Kendall Lauber, Teddy Aloo, Ben Collins, Edwin Gumbe, K Rivet Amico, Kennedy Olango, Wilson Odero, Susan M. Graham

**Affiliations:** 1 Department of Health Behavior and Health Education, School of Public Health, University of Michigan, 1415 Washington Heights, SPH I, Ann Arbor, MI 48109, USA;; 2 Department of Epidemiology, Mailman School of Public Health, Columbia University, 722 W 168th St., New York, NY 10032, USA;; 3 Anza Mapema Tom Mboya Center, Nyanza Reproductive Health Society, Kisumu 40100, Kenya;; 4 Men Against AIDS Youth Group (MAAYGO), Milimani Box 1174, Kisumu 40100, Kenya;; 5 Department of Family Medicine and Community Health, School of Medicine, Maseno University, Private Bag, Maseno 40105, Kenya;; 6 Departments of Global Health and Medicine, University of Washington, Box 359909, 325 Ninth Avenue, Seattle, WA 98104, USA;

**Keywords:** resilience, gay, bisexual, male youth, qualitative

## Abstract

Gay and bisexual male youth in Kenya experience human rights violations, including pervasive stigma and discrimination, and these oppressive forces are associated with elevated rates of mental health concerns. Despite these challenges, many gay and bisexual male youth in Kenya are thriving during this critical developmental period. This study explored intrapersonal processes that gay and bisexual male youth in Kisumu, Kenya, highlight as important to developing, and demonstrating resilience in the face of adversity. We conducted qualitative in-depth interviews (IDIs) with 40 gay and bisexual male youth, ages 20–30 (mean = 26.4), and an additional 20 IDIs with gay and bisexual men, ages 22–45 (mean = 26.6), who were working as peer educators (total *n* = 60), all in Kisumu, Kenya. A total of nine primary themes emerged which describe various intrapersonal resilience processes enacted by gay and bisexual male youth, including sexual identity acceptance, self-confidence, self-love, religious/spiritual affirmation, adaptive coping, successful navigation, legal rights awareness, economic stability, and advocacy satisfaction. These data demonstrate the range of positive personal processes that promote mental health and wellbeing among gay and bisexual male youth in Kenya. We discuss implications of these findings for community-based interventions, and call for a research paradigm shift away from deficits and toward resilience.

## 1. Introduction

Sexual and gender minority (SGM) people in Kenya experience multiple forms of human rights violations, such as verbal and physical violence; rape and sexual assault; denial of healthcare; and legal and cultural barriers to relationships, housing, education, and employment [1–8]. Much of the stigma and discrimination that fuels such violations in Kenya, and elsewhere in sub-Saharan Africa, stem from outdated colonial laws that criminalize same-sex behaviors, and conservative religious beliefs brought to the continent by European colonizers [9–11]. Prior to widespread dismantling of local cultural practices and the colonization of most regions of sub-Saharan Africa by Europeans, there were multiple forms of culturally sanctioned and socially accepted same-sex behaviors, identities, and relationships across the African continent [12–14]. Specifically in Kenya, there are well-documented examples of same-sex relationships and partnerships within various ethnic tribes, and growing numbers of scholars and activists are calling for the rejection of oppressive laws and policies against SGM people that are remnants of colonial oppression [5,9,15–17].

While studies specifically focused on SGM human rights violations and their psychological sequela in Kenya are sparse (for exceptions see [1,18]), there are an increasing number of studies focused on HIV related issues among gay and bisexual men, and other men who have sex with men in Kenya that include data on experiences of violence and discrimination. One report from the Anza Mapema study in Western Kenya found that 39.1% of their mixed HIV serostatus sample reported recent physical or psychological trauma, whereas another report focused just on the HIV-negative men in their sample and found that 51.5% reported such experiences [4,19]. In a study conducted in the coastal region of Kenya, 66.9% of 112 gay and bisexual men and other men who have sex with men reported forced or coerced sex, physical abuse, emotional abuse, threats or intimidation related to their same-sex behavior within the past year [2], whereas a study of 595 gay and bisexual men, other men who have sex with men, and male sex workers in central Kenya (Nairobi) found that 47.4% of their sample had experienced the same types of recent trauma [20]. In a three-site combined dataset of 1476 gay and bisexual men and other men who have sex with men in three regions of Kenya (Central, Western, Coast), slightly more than half (51.2%) of the participants reported recent trauma or abuse related to same-sex behavior [3].

An examination of the age ranges of prior studies examining experiences of trauma and abuse among gay and bisexual men in Kenya reveals that many of these samples are comprised of young men under the age of 30. In the three-site combined dataset of 1476 gay and bisexual men in the three regions of Kenya previously mentioned, the median age was 25 years, with an interquartile range of 22 to 29 years, and in the Nairobi study of gay and bisexual men and male sex workers, the median age was 27.2 with a standard deviation of 7.2 [3]. This is relevant since the 2010 Kenya Constitution defines “youth” as a person aged 18 years and who has not reached the age of 35 years. The Kenya Youth Development Policy 2019 [21] adopts this constitutional definition of youth, but includes individuals down to age 15, which is in alignment with the United Nations and World Health Organization’s conceptualization of youth as including individuals between the ages of 15–24 [22,23]. The Kenyan Youth Development policy conceptualizes the period of “youth-hood” as including many of the developmental trajectories and challenges that are often described during adolescence and emerging adulthood in the United States [24–26], and views this time period as one of many transitions and vulnerabilities [21].

In Kenya, youth constitute a significant share of the country’s population, and a considerable number of studies have demonstrated persistent risks and challenges faced by Kenyan youth, calling for the need to develop and implement appropriate strategies, policies and programs to mitigate these risks and challenges [27]. Unfortunately, conducting research in Kenya with gay and bisexual male youth under the age of 18 is generally prohibited due to concerns that such research may encourage young people to engage in same-sex behavior, which is prohibited by law. In order to be in alignment with cultural conceptualizations of human development in Kenya, this paper will adopt the definition of youth as ages 18–30, inclusive.

### Minority Stress Model and Resilience

The pervasive stigma and discrimination experienced by gay and bisexual male youth in Kenya has been shown to be associated with increased psychological distress and mental health concerns [2,3,18]. This is in alignment with the minority stress model, which purports that pervasive stigma and discrimination against SGM people results in experiences of unique stressors, which, coupled with general life stressors, can lead to negative physical and mental health outcomes [28]. This model differentiates between distal stressors, which are events and experiences outside the person (e.g., life events, discrimination, microaggressions), and proximal stressors, which are internalized cognitive processes in reaction to discriminatory societal forces (e.g., internalized homophobia, expectations of rejection, concealment of sexual identity) [28,29]. The minority stress model also demonstrates that both coping and social support can decrease the negative effects of the stressors so that negative health outcomes can be reduced, or completely avoided altogether, when these protective factors are in play.

The minority stress model is grounded in extensive theory and empirical research, demonstrating how adversity and stress are critical factors in the development of negative health outcomes [30–33]. Although the initial development and empirical research supporting the minority stress model was conducted in the United States, the utility of this model has been demonstrated with populations of SGM people in Kenya [18], as well as with gay and bisexual youth and men in several other countries in sub-Saharan Africa, such as Nigeria [34,35], South Africa [36,37], and Zambia [38].

Resilience is embedded within the minority stress model and has been defined in a variety of different ways. Often it is viewed as a dynamic process whereby a person is able to positively adapt and thrive despite risk exposure, high levels of stress, and other forms of adversity [39–43]. In addition to the presence of risk, some scholars suggest that, in order for resilience to occur, there must be the presence of promotive factors, which assist in the process of reducing or avoiding risk [39,43]. Fergus and Zimmerman’s (2005) conceptualization of resilience among adolescents has identified two types of promotive factors—assets and resources. They view assets as positive factors that are internal to the individual and include attributes such as self-efficacy, high self-esteem, competency, and coping skills. Resources, on the other hand, are factors that are external to the individual and include influences such as parental support, natural mentors, community-based organizations, and youth development programs. Prior qualitative and quantitative studies with Black American gay and bisexual male youth have demonstrated the presence of both assets and resources in the resilience processes demonstrated by this sexual minority group [44–46].

In the context of the minority stress model, the presence of resilience processes can decrease the negative impact of minority stress on health and lead to more positive adaptation to such stress and subsequent improved health outcomes [47,48]. Meyer (2015) delineates that, although resilience is similar to coping given they both can buffer the negative effect of stress on health, coping refers to efforts the person makes to adapt to stress, regardless of whether or not those attempts are successful. Resilience, on the other hand, indicates success in adaptive functioning in the face of stress. He has suggested that there are two types of resilience: individual-based resilience, which is focused on personal agency and mastery, and community-based resilience, which is focused on connectedness to community and social resources [47]. There are places of intersection of these two types of resilience, such as with social identity as an LGBTQ+ person (individual) offering entre to LGBTQ+ community resources (community). Those who do not identify with the larger LGBTQ+ community will not likely have access or invitations to the range of resources that exist within community organizations and spaces and, furthermore, will not gain beneficial social support from interactions with other sexual and gender minority people [20].

Examinations of resilience processes among gay and bisexual male youth in Kenya are limited, and the few that do exist have measured resilience in different ways. Harper et al. (2015) explored a range of intrapersonal and interpersonal factors associated with the resilient outcomes of sexual health (i.e., condom use and HIV testing) and psychosocial health (i.e., high self-esteem and psychosocial wellbeing) among 511 gay and bisexual male youth in Western Kenya and found association across a range of factors. Their focus was not on understanding the process of resilience, but instead factors associated with resilient outcomes [49]. Similarly, Ogunbajo et al. (2019) examined interpersonal and intrapersonal promotive factors, such as self-esteem, psychological wellbeing, social support, and membership in LGBTQ+ organizations, in the context of pre-exposure prophylaxis (PrEP) awareness and acceptability, although they did not explicitly name these factors as resilience processes [50]. In one of the only qualitative studies that addresses resilience processes among gay and bisexual men in Kenya, Graham et al. (2018) conducted 30 qualitative interviews with men living with HIV about antiretroviral therapy (ART) and HIV care (median age = 31, range 19–51). They found that resilience emerged as an important factor in facilitating men’s motivation for HIV care engagement, ART adherence, and living a healthy and fulfilling life, and conceptualized it as primarily an individual-level factor [51]. Since their focus was on ART and HIV care, they did not have data to conduct a more in-depth analysis of resilience.

Despite the many challenges that confront gay and bisexual male youth in Kenya, many are surviving and thriving during this critical developmental period, as evidenced by an increasingly more visible SGM community presence with SGM specific community-based organizations and local, regional, and national advocacy efforts [9,20,52]. In addition, as previously detailed, resilience can decrease the negative impact of minority stress on health, and lead to more positive adaptation to such stress and improved health outcomes [47]. Given the accumulating evidence of psychosocial distress and mental health challenges experienced by gay and bisexual male youth in reaction to pervasive SGM stigma and discrimination in Kenya [2,3,18], it is important to gain a better understanding of how resilience processes can function to ameliorate this relationship. Qualitative research that allows participants to share their stories of resilience in the face of adversity is needed in order to have a more nuanced understanding of this strengths-based process. Focusing on the strengths of gay and bisexual male youth in Kenya will also intentionally oppose the dominant perspective of sub-Saharan African SGM populations as victims rather than communities in which strong resilience and resistance in the face of extreme oppression is constantly demonstrated.

The focus of the current analysis is on gaining a deeper understanding of the array of individual-level resilience processes used by gay and bisexual male youth living in Kisumu, Kenya. Therefore, our overarching research question is as follows: what types of intrapersonal resilience processes are enacted by gay and bisexual male youth in Kisumu, Kenya, as they strive to develop and demonstrate resilience in the face of adversity and oppression? By conducting qualitative individual in-depth interviews with gay and bisexual male youth, as well as gay and bisexual peer educators who were slightly older, we were able to center male youth experiences and voices.

## 2. Materials and Methods

### 2.1. Participants

The data for the current study came from a larger parent study focused on the development and evaluation of a PrEP and sexual health promotion intervention for gay and bisexual male youth in Kisumu, Kenya. Since the parent study sought to learn from participants with a range of PrEP experiences, we utilized a 2 (PrEP experience) × 2 (PrEP interest) stratified purposive sampling frame to recruit 40 HIV-negative gay and bisexual male youth between the ages of 18–30 years, with varying levels of PrEP experience and interest for individual in-depth interviews (IDIs). We refer to these participants as Community Members. This resulted in 10 PrEP experienced participants currently taking PrEP, 10 PrEP experienced participants who have stopped taking PrEP, 11 PrEP naïve participants interested in taking PrEP, and 9 PrEP naïve participants who have no interest in taking PrEP. We included gay and bisexual male youth of various ages within each stratification cell. In addition to the 40 gay and bisexual male youth, we conducted 20 IDIs with gay and bisexual men who were working as peer educators in HIV testing and PrEP programs in the Kisumu area. We refer to these participants as Peer Educators.

Community Member participants met the following inclusion criteria: biologically male at birth and currently identify as a man, ages 18–30 inclusive, resident of Kisumu, reported at least 1 act of anal or oral intercourse in the previous 6 months with another man, self-reported as not living with HIV, and willing and able to provide informed consent and participate in an IDI. Peer Educator participants had all of the same inclusion criteria except HIV status and age, and the additional criteria of currently working as a Peer Educator or similar role in an HIV testing or PrEP program in the Kisumu area. Overall, we sought to recruit individuals who were perceived to be good key informants, defined as a person who thinks about the study topics, is comfortable talking about these topics, and is good at describing their thoughts and feelings.

Demographics of our sample are provided in Table 1. Community Members ranged in age from 20 to 30 (mean = 26.35) and the majority identified as bisexual (47.5%), whereas Peer Educators ranged in age from 22 to 45 (mean = 26.6) and the majority identified as gay and bisexual (both 35%). The majority of Community Members had attained a diploma and were working part-time, while Peer Educators had completed secondary school and were working part-time. The majority of both groups were Christian and Luo, and the majority of Peer Educators had been working as such between 1 and 2 years.

### 2.2. Qualitative Interview Guide

Research team members with extensive experience working with gay and bisexual male youth in Kenya created a semi-structured qualitative interview guide for the parent study with significant input and guidance from the qualitative interviewers, who were all gay or bisexually identified men from Western Kenya. Throughout the course of qualitative interviewer training, modifications were made to the guide to assure its utility with gay and bisexual male youth in Kisumu. The interview guide was grounded in phenomenological and constructivist frameworks, which provided a general structure for discussion but required participants to provide their own conceptualizations of terms and phrases based on their lived experiences. The guide included a series of questions and probes focused on 4 primary areas based on the parent study’s research questions: health issues affecting gay and bisexual male youth, thriving/coping as a gay or bisexual male youth, experiences with PrEP, and recommendations for improving PrEP services for gay and bisexual male youth. The structure and content of the questions did not follow any prior theory or framework, thus we were able to conduct an inductive inquiry of participants’ thoughts, feelings, and experiences in these general areas, as described by the participants.

### 2.3. Procedures

Given the purposive nature of our sampling frame, we recruited participants through outreach activities conducted by our interviewers at community-based organizations (CBOs) and health clinics in Kisumu, who provide services to gay and bisexual men and youth. Our research team has worked with these CBOs and clinics for more than 10 years, and conducted participant recruitment for other gay and bisexual men focused studies using similar procedures in these venues. Recruitment and screening took place verbally with gay and bisexual male youth who fit criteria in accordance with our inclusion criteria and stratified sampling framework. All interviewers followed safety protocols and no specific recruitment materials were used in order to provide a greater degree of safety and security for interviewers and potential participants.

Interviews took place in private rooms at 1 of our CBO or clinic research sites. Interviewers first obtained verbal consent for research participation and then verbally administered a brief demographic survey. They then conducted the IDI and recorded it using a digital audio recorder. Following the interview, the interviewer debriefed with the participant and then provided him with a monetary incentive and a resource guide that provided information on an array of physical and mental health services that are friendly to gay and bisexual men and youth. The interviewer then completed a written post-interview summary that offered details in the following areas: (a) overall impressions, (b) key information provided, (c) PrEP specific information, (d) participant recommendations, and (e) recommendations.

Interviews were conducted in a mix of English, Dholuo, and/or Kiswahili, based on the most comfortable language for the participant (all interviewers were fluent in all 3 languages). Recordings were simultaneously translated and transcribed by a local transcriptionist in Kisumu who has extensive experience with simultaneous translation/transcription for interviews conducted with gay and bisexual young men. All transcripts were de-identified and quality checked by team members to ensure accuracy of transcription. The Institutional Review Boards of the participating U.S. academic institutions, as well as the Ethics Review Committee of our local Kenyan academic partner, approved all study procedures.

### 2.4. Data Credibility and Analysis

Several procedures were put into place during data collection and analysis to increase the credibility of our findings, thus increasing alignment between the perspectives shared by participants and our representation of those perspectives [53,54]. In alignment with Lincoln and Guba’s (1985) recommendations, we implemented the following credibility strategies: prolonged engagement, persistent observation, triangulation, and member checking [53]. With regard to prolonged engagement and persistent observation, senior members of our research team have worked with the broader SGM community in Kisumu for more than 10 years, with a primary focus on gay and bisexual male youth. These interactions have not only included research activities, but also capacity sharing and program development activities, social and cultural events, and community organizing. In addition, some research team members have provided medical and mental health care and services to gay and bisexual men and youth in the community through local community clinics and CBOs. Through these in-depth and prolonged interactions and activities, the research team has gained a high degree of trust with members of the community. We also implemented various types of triangulation by having 5 different interviewers collect data, collecting data from 2 different groups of key informants (Community Members and Peer Educators), and working with 6 different analysts at varying educational levels and disciplinary backgrounds (public health and psychology). Finally, we engaged in member checking by presenting the results to 4 of the 5 interviewers (all gay and bisexual male youth or men from Kisumu) and having them provide feedback and confirmation regarding the analytic findings.

The current analysis sought to explore the various ways in which gay and bisexual male youth in Kenya demonstrate resilience processes despite pervasive stigma and discrimination against sexual minorities throughout the country. Since the focus of this study is on the lived experiences of gay and bisexual male youth in Kenya, we conducted the analyses using a phenomenological inquiry framework [55,56]. Phenomenology is specifically focused on describing what a given group of people have in common as they experience the same or similar experiences or phenomena, and is an inductive analytic approach that allows the patterns, themes, and categories of analysis to emerge from the voices of participants. The composite descriptions of the phenomena of resilience processes presented in this article explain the underlying structure that exists across participants [56,57]. In line with our phenomenological framework, we conducted the analyses to assure that different voices were represented in the findings and that dominant perspectives did not silence conceptual “outliers”. Thus, all voiced themes are presented instead of only those that were endorsed by a majority of participants [56,57].

The analyses were conducted by a group of 6 analysts from the U.S., representing educational levels from undergraduate students to graduate students, to a master’s level staff member, and a doctoral level faculty member. Both the staff and faculty member have extensive experience working in Kenya, are primary investigators on the parent study, and trained and supervised the interviewers in Kenya. All student analysts were given required background reading to familiarize themselves with both the study population and qualitative analysis. In addition, analysts read all 60 of the post-interview summaries that were provided by the interviewers in order to gain an understanding of the nature and overall findings of the interviews.

We used an inductive consensus building process to determine the focus of the current analysis. In addition to the 60 post-interview summaries, each member read 5 transcripts to brainstorm questions and common themes in margin notes, with each person overlapping on only 1 transcript with another analyst. The team met on a weekly basis to discuss the content of the transcripts and to build consensus on an area of focus for the analysis. Once a broad research question was developed (what do resilience processes look like across socioecological levels for gay and bisexual male youth in Kisumu Kenya?), subsequent meetings involved reading more transcripts, reviewing notes to recognize key themes, selecting key levels of support (intrapersonal, interpersonal, organizational, communal), and developing a formal codebook to name and operationally define each code.

All transcripts were then divided evenly between team members, ensuring variability and overlap. At least 2 different analysts reviewed each transcript in order to increase analytic dependability. Team members then participated in open coding which involved applying codes to their assigned transcripts, and noting key representative quotes. As coding progressed, the team met weekly to review the codes and make modifications to the codebook, which involved eliminating, collapsing or splitting codes. Discrepancies in coding were resolved through discussion and consensus building. Given the amount of data analyzed and the depth of the findings, in order to address our broad research question, we divided the results into separate papers. The focus of the current analysis is on intrapersonal resilience processes demonstrated by gay and bisexual male youth in Kisumu, Kenya.

## 3. Results

A total of nine primary themes emerged, which describe various intrapersonal resilience processes enacted by gay and bisexual male youth in Western Kenya. The primary themes included the following: sexual identity acceptance, self-confidence, self-love, religious/spiritual affirmation, adaptive coping, successful navigation, legal rights awareness, economic stability, and advocacy satisfaction. In the following paragraphs, we describe the themes and sub-themes, and offer representative quotes to further illustrate the themes. We also provide a pseudonym for each participant quote, including information regarding their age, self-identified sexual orientation, and whether they are a Community Member or Peer Educator.

### 3.1. Sexual Identity Acceptance

Sexual identity acceptance is a sense of acceptance and/or pride in one’s sexual orientation. These sentiments were often coupled with discussions about how sexuality is innate, is not a curse, cannot be denied, and is a crucial part of one’s identity. Participants also discussed a sense of peace and comfort they felt from accepting their own sexual identity and sharing it with others, such as enjoying being in a same-sex relationship. Below, Hermen describes his personal history of self-acceptance, while Tony describes his recognition that his sexual identity does not place him apart and that he is equal to all others in Kenya, who, at the end of the day, all use the same resources/facilities.

“Okay what has helped me to not have issues with myself is the fact that I accepted myself a long time ago and I convinced myself that I tried to conform with the society by trying to date and all that but it didn’t work, so I convinced myself a long time ago that am not going to live a lie, am going to live my life like it is and this is my life. So I convinced myself, I accepted myself and I am now comfortable in my own skin, so I don’t have issues.”

Hermen, 23, Gay, Peer Educator

“I see myself from a different perspective and angle because one I am a Kenyan, when ah other people who think they are straight go to the bank I use the same bank hall … we use the same motor vehicle, taxis, you know it is a free world for everybody so I got no time to think of myself lesser than others.”

Tony, 26, Gay, Community Member

### 3.2. Self-Confidence

Self-confidence is having confidence in one’s ability to be successful in life and achieve one’s goals. Participants expressed satisfaction in their achievements, their determination to succeed, and their ability to take care of themselves (and, in some cases, others) without relying on others. Self-confidence was also a protective factor against rejection. Participants were determined to and confident in achieving their goals, despite the challenges they faced. Below, Salim describes the importance of believing in himself, while Suleiman describes his self-sufficiency.

“(Interviewer: what has helped you to survive?) So many things … believing in myself. If you believe yourself that you can … even if people talk shit about you, you still have to move on … whatever I set on my mind I don’t have bad attitudes towards it. I keep it and working on it.”

Salim, 28, Gay, Peer Educator

“I am a hard working person and I like doing my things, I don’t depend on people, I don’t even depend on family … even if my family rejected me that is their own cup of tea because where I have reached now. I am a sure Kenyan citizen, I can take care of myself and I can take care of other people, so if you want to reject me, fuck you that is your own business. I am moving on and you might reject me but other people accept me.”

Suleiman, 29, Gay, Community Member

### 3.3. Self-Love

Self-love is a state of overall self-acceptance and feelings of extreme value and self-worth. Self-love described sentiments similar to the sexual identity acceptance and self-confidence themes above, but extended to a deeper and more emotional level. Participants who were and were not open with others about their sexual orientation expressed self-love. They discussed loving their sexual orientation and loving themselves as a gay or bisexual male youth. Self-love led to self-awareness that served as a protective factor against LGBTQ+-specific discrimination and rejection. Below, Peter describes the communal and personal benefits he has gained from loving himself, while Michael describes his personal journey to self-love.

“I like it [being gay] because that is who I am, I love myself like that… it has brought me close to people of my community, it has made me to know my status and how to protect myself.”

Peter, 25, Bisexual, Community Member

“Yah like aah before then, I never liked anything about me. But now, I seriously like everything about me. Everything I do, every step I take just like it even if other people don’t like it. I just feel is a … is me. I just like being me.”

Michael, 26, Gay, Community Member

### 3.4. Religious/Spiritual Affirmation

Religious and spiritual affirmation is support coming from one’s belief and trust that they are loved by God. It is important to note that this factor promotes resilience on the intrapersonal level, while often invoking negative experiences on the interpersonal and political levels. This internal affirmation came from the participant’s belief and trust in a non-judgmental, accepting God who created LGBTQ+ people as equals, as described by Tony, a 26-year-old gay Peer Educator, “God didn’t make a mistake in creating me.” This is further demonstrated by Moses’ description of his personal creation by God and Feshal’s recognition that God created everyone including gay people.

“I believe in myself … I care less on what people uuh saying about me which I believe to be negative … I also believe I’m a child of God … and God also created me with a purpose … I know I exist as any other person.”

Moses, 28, Bisexual, Community Member

“If only these people could just live their life like God’s intention- I don’t believe this thing [being gay] that it is un-Christian … God is the one who created everyone and God is the one who created gays, so live your life as a gay person, there is no need of hiding.”

Feshal, 30, Gay, Community Member

### 3.5. Adaptive Coping

Adaptive coping involves enjoyable activities that help relieve day-to-day as well as LGBTQ+-specific stress. Participants described three major categories of adaptive coping strategies: physical activities (e.g., swimming, soccer, dancing, jogging), creating art (e.g., playing music, working in a studio, participating in performing arts, writing), and consuming media (e.g., reading, watching TV/movies/sports games). Participants discussed the joy and stress relief they get from participating in these activities. Below, Augustine describes various coping strategies, and Wickliffe describes writing stories as a healthy form of meditation.

“What I like doing like playing football I always feel good because I am stress free, at that time I feel like there is nothing am thinking about am just playing ball and enjoying it … I can play music, listen to music, maybe talk to friends … I can also go to [LGBTQ+ drop in center] and sit with the colleagues and watch TV so I feel good because they have got a safe space there.”

Augustine, 24, Bisexual, Community Member

“ … when I feel like tormented by other people, when I feel like I have been oppressed by other people, when my feelings have been touched … I will say that is the time now … [to] do what I love doing that is writing, yeah, that is the time I will be seated somewhere writing stories, and now it always influence me in this way, whatever I have gone through … it is like a form of meditation.”

Wickliffe, 20, Gay, Community Member

### 3.6. Successful Navigation

Successful navigation refers to moving through the world and interacting with others in order to reflect one’s true self, while also protecting one’s physical safety given pervasive anti-LGBTQ+ stigma. Participants often talked about using forms of code switching, a process by which an individual changes their demeanor based on their changed setting. While one may be open about their sexuality with LGBTQ+ friends/peers, they may conceal it with the general population by changing the way they carry themselves, talk, walk or dress. Below, Duncan and Nicholas describe how being acutely aware and properly responding to their setting is a necessary asset to self-preservation.

“ … self-respect … helps me … know where to express myself and where not to express myself openly because security begins with me.”

Duncan, 23, Bisexual, Community Member

“I am prone to risks, and ah security threats, so being able to assess the environment and being able to be sensitive on the surrounding, so that I can dance to the tune of whoever wants to watch or see what, so if it is dressing you dress carefully, you watch your mouth, you watch your steps, you watch where you go, where you party, such like things, so just being aware of the environment.”

Nicholas, 27, Bisexual, Peer Educator

### 3.7. Legal Rights Awareness

Legal rights awareness refers to an understanding of the Kenyan legal system and constitution as it pertains to one’s protections from harassment and discrimination. Participants reported learning their rights from sources such as school, LGBTQ+ organizations, and sensitization campaigns, and using them to protect against LGBTQ+-specific harassment, rejection from healthcare facilities, and/or physical violence. Below, Duncan describes learning his rights in school, while Abdi discusses this knowledge as a form of self-empowerment.

“One I-I think I think I thank my folks for taking me to school so that then means I know some of my rights, I might not know all because I am not a lawyer but then I know the basics that protect me.”

Duncan 23, Bisexual, Community Member

“I am empowered, ah I know what my rights are, I can be violated when I am quiet because I fear what people will say, I will always speak my mind.”

Abdi, 25, MSM, Community Member

### 3.8. Economic Stability

Economic stability is the state of being financially secure so that one can comfortably afford needs such as healthcare, rent, and food, as well as desired commodities (e.g., the ability to go out with friends or support others). Economic stability has a strong impact on participants’ physical, mental, and sexual health as it hinders their sense of independence and self-confidence. Therefore, it was frequently discussed as a protective factor against LGBTQ+ discrimination. For example, a participant who financially supported their family was less likely to be rejected, as their economic power placed them as the household decision maker. Below, Bonny describes how financially supporting his family detracts from their focus on his sexual orientation, and Geoffrey describes the impact of financial security on his mental health.

“ … being economically fit … puts you in a- in a position in a society that not everyone would question your sexual orientation. If you’re in a position to provide for your dad for your mom for your siblings, then who is there to complain or to doubt your sexual orientation? Because we are living in a society where the moment you don’t provide, you are the talk of the town. The moment you provide, everyone knows that you are good … I support my family in one way or another, and this, this really puts me in a good position in the family, so they feel like I’m responsible and that is what they use to judge me, they don’t use my sexual orientation to judge me.”

Bonny, 28, Bisexual, Peer Educator

“How poorly I feel mentally is just the financial part of it, I believe that is one that cut across, when I am financially down I normally feel like ah- … feel like I do not deserve to live, so financial problem is there for poorly function of the brain or mental health.”

Geoffrey, 23, Bisexual, Peer Educator

### 3.9. Advocacy Satisfaction

Advocacy satisfaction at the individual level refers to the sense of gratification or joy that one experiences by giving back to their community. Participants expressed feeling fulfilled from a range of activities, including teaching others about their rights, volunteering, visiting community members in hospitals, working as a peer educator, and referring others to healthcare facilities. By taking part in these activities, participants felt they were a part of a larger movement. Below, Newton describes educating and sensitizing those who are not part of the LGBTQ+ community, and Richard describes using his own story to create change in his community.

“I have used my … knowledge of things I know about the GBMSM … to also sensitize those who aren’t gay … others have accepted, others are still accepting, others haven’t, [I] am still doing my work by reaching out to them, giving the information as much as I can so that they are able to accept. So that has been my greatest- my greatest sort of coping mechanism.”

Newton, 28, Gay, Community Member

“I was also kind of one of the victims who I happened to be-okay I got raped maybe when I was young, so I found myself wanting to do these activities to get this training on health works so I can also make my community aware on such issues.”

Richard, 29, MSM, Peer Educator

## 4. Discussion

We used qualitative interviews to better understand intrapersonal resilience processes enacted by gay and bisexual male youth in the face of SGM-focused oppression and discrimination, which supported their mental health and wellbeing. These resilience processes surfaced when participants discussed their sexual minority identities and the ways that others reacted to those identities in everyday life, as well as healthcare specific settings. The nine themes that emerged were sexual identity acceptance, self-confidence, self-love, religious/spiritual affirmation, adaptive coping, successful navigation, legal rights awareness, economic stability, and advocacy satisfaction. In the face of oppression and discrimination, these positive intrapersonal resilience processes were critical in promoting the holistic physical and mental wellbeing of the gay and bisexual male youth in Kenya.

Because the literature that exists on this population mostly examines how negative factors impede physical and mental wellbeing, we intended to identify positive factors that facilitate physical and mental wellbeing. First, we rooted our analysis in the Kenyan context. We noted that stigma and discrimination impact the mental health of these youth as described in the minority stress model. Distal stressors included discrimination from Kenyan law, institutions (e.g., occupational settings), and communities (e.g., families, neighbors), and proximal stressors included internalized stigma and concealment of sexual identity [29]. These stressors are complex, vary widely within the Kenyan context, and impede positive mental health. We then aimed to conceptualize what resilience processes gay and bisexual male youth in Kenya enacted to survive and thrive. The nine key themes described above were used by these youth as individual-level processes to counter exposure to emotional and physical risk, and to promote positive mental health [29]. These themes represent what Fergus and Zimmerman (2005) describe as intrapersonal assets [39]. These processes can be individually and collectively leveraged to promote positive wellbeing of gay and bisexual male youth.

### 4.1. Implications for Intervention

Moving forward, we hope to encourage the development of interventions that increase resilience among gay and bisexual male youth at multiple levels: interpersonal, community, and political. At the interpersonal level, we recommend promoting self-growth through one-on-one discussions. This would allow gay and bisexual male youth to share their stories of self-love, as well as discuss various coping strategies for survival and successful navigation. Interpersonal interventions would be twofold, by allowing those who speak to become role models and audible advocates, and those who listen to learn and feel hopeful. With time, this could evolve to a ripple effect, leading listeners to become speakers as the communities grow. By breaking down the barriers of stigma within gay and bisexual male youths’ interpersonal relationships, these youth can feel supported in their personal journeys towards self-confidence, self-acceptance, and self-love. It will be beneficial to identify both the most effective and safe way to share this information with other gay and bisexual male youth (e.g., social media campaigns, dramas, spoken word, interactive presentations), and the most acceptable and trusted people to disseminate this vital information to other gay and bisexual male youth, especially given the sensitive nature of same-sex sexuality in Kenya.

At the community level, we suggest education and active involvement of the larger communities within which gay and bisexual male youth live. Education must involve discussion of gay and bisexual male youths’ struggles and laws that infringe on their human rights. In this way, we can touch the minds and hearts of Kenyans to promote the growth of communities that support and include gay and bisexual male youth. This can be done through sensitization training to normalize and humanize gay and bisexual male youth, discussion forums to facilitate active dialogue about necessary political change, and social media campaigns that promote intrapersonal reliance and inspire community support. Additionally, for community members who view gay and bisexual male youth as sexual figures and vectors of disease transmission, we need to find ways to help them see that these youth are valuable contributors to Kenya’s society and economy.

This mindset shift can grow the number of allies, create safe spaces, and increase assistance and donations for gay and bisexual male youth and the organizations that support them. This communal acceptance can be facilitated within peer groups, families, neighborhoods, and religious groups. Although some gay and bisexual male youth in Kenya become disconnected from their churches due to anti-gay Biblical teachings, quite a few of our participants mentioned religion and spirituality as resilience factors. Therefore, targeted interventions could be used to sensitize religious leaders and counter the spread of anti-gay teachings. This could leverage the religious community sphere (e.g., churches, Bible study groups) to promote positive dialogue about gay and bisexual male youth and facilitate religious and spiritual affirmation.

Dissemination of these data to several key stakeholders will be important in order to promote an increased focus on promoting the mental health and wellbeing of gay and bisexual male youth in Kenya through the promotion and support of resilience processes. This information can be used to improve community-based healthcare services by educating providers on the range of resilience processes utilized by some gay and bisexual male youth, so they can encourage and support their gay and bisexual male youth clients in the use of these strengths-based approaches for self-care. Such information would also be valuable for professionals working in organizations and institutions that provide any type of services or support to gay and bisexual male youth, including schools and places of worship. Community-based organizations that provide specific programming for gay and bisexual male youth can use these data in their strategic planning processes, including decisions about thematic focal areas and program decision making. Finally, these data can be shared with international and local donor partners to encourage them to have a greater focus on the mental health and wellbeing of gay and bisexual male youth, and to create and maintain funding streams for grants that support resilience focused and strengths-based approaches to service provision for gay and bisexual male youth.

At the governmental and political level, findings from this study could be shared with the National AIDS and STIs Control Programme (NASCOP) and the Key Population Technical Working Group (KPTWG) to assist with program planning for gay and bisexual male youth, as well as the Kenyan Ministry of Health as they focus on the objectives and strategies outlined in the 2015–2030 Kenya Mental Health Policy. We must encourage the Kenyan government to prioritize the physical and mental health of gay and bisexual male youth. This can be done through the enactment of SGM occupational and social protections, which would help to facilitate social acceptance and economic stability for gay and bisexual male youth. In addition, despite the Kenya High Court’s decision in May 2019 to reject a petition that would have declared Sections 162 and 165 of the Kenya Penal Code unconstitutional, thus decriminalizing same-sex behavior among consenting adults, advocacy for the reintroduction and passage of this petition is needed in order to support the mental health and wellbeing of gay and bisexual male youth.

### 4.2. Future Research, Strengths, Limitations

Our findings also hope to promote a paradigm shift in future research with gay and bisexual male youth in Kenya from a deficit-focused approach to a strength-based framework. More primary studies that focus on identifying and promoting strength and resilience factors and processes among gay and bisexual male youth in Kenya are needed. These studies should aim to conceptualize resilience factors for holistic overall health, as existing literature tends to focus on sexual health (e.g., PrEP, condom usage). Additionally, because tribes across Kenya have differing cultural values, beliefs, and practices, we must recognize how young gay and bisexual male youths’ lived experiences differ by tribe and geographic location, and modify our resilience promoting studies and interventions to fit their cultural contexts. This would include attention to the use of geographically-specific languages, as well as the use of materials that are accessible to those with low to no literacy. More data collection from central Kenya, coastal Kenya, and rural areas of Kenya is needed to understand the breadth of intrapersonal resilience factors.

The primary strengths of this study lie in its methodological qualitative inquiry that centered community voices through open-ended qualitative interviews. Recruitment was done throughout the community, resulting in a large sample size and breadth of data from 60 different gay and bisexual male youth and men. Interviews were conducted by trained and trusted community members and advocates who identified as gay and bisexual men, all of whom have had long-term connections to the larger research team. This promoted safe and trusting conversations that centered community voices. In the analysis stage, the use of six analysts allowed for collaborative study investigation and cross checking. Further, the involvement of the Kenyan interviewers in member checking the findings from the analyses, contributed to the credibility of these findings. Limitations include the secondary analysis nature of this study, as data came from a larger study focused on developing and evaluating a PrEP and sexual health intervention. This may have led to sampling bias, as recruitment was rooted in PrEP interest (though purposive sampling recruited both those interested and not interested in PrEP), and the Community Member sample was limited to HIV-negative gay and bisexual male youth. Furthermore, we acknowledge that none of the original analysts were Kenyan.

## Figures and Tables

**Table 1.** Sample demographics.

	Community Members (*n* = 40)	Peer Educators (*n* = 20)	Combined (*n* = 60)
Age	Mean = 26.35 years (range: 20–30)	Mean = 26.6 years (range: 22–45)	Mean = 26.4 years (range: 20–45)
Sexual Orientation			
Gay	16 (40.0%)	7 (35.0%)	23 (38.3%)
Bisexual	19 (47.5%)	7 (35.0%)	26 (43.3%)
MSM	5 (12.5%)	4 (20.0%)	9 (15.0%)
Other	0 (0%)	2 (10.0%)	2 (3.3%)
Highest educational level			
Primary School	1 (2.5%)	1 (5.0%)	2 (3.3%)
Secondary School	11 (27.5%)	7 (35.0%)	18 (30.0%)
Certificate	6 (15.0%)	5 (25.0%)	11 (18.3%)
Diploma	15 (37.5%)	5 (25.0%)	20 (33.3%)
Bachelor’s Degree	4 (10.0%)	0 (0%)	4 (6.7%)
Master’s Degree	0 (0%)	1 (5.0%)	1 (1.7%)
Currently attending school	3 (7.5%)	1 (5.0%)	4 (6.7%)
Current Employment			
Part-time	16 (40.0%)	15 (75.0%)	31 (51.7%)
Full-time	4 (10.0%)	1 (5.0%)	5 (8.3%)
Casual Laborer	5 (12.5%)	0 (0%)	5 (8.3%)
Sex worker	2 (5.0%)	2 (10.0%)	4 (6.7%)
Not working/in school	3 (7.5%)	0 (0%)	3 (5.0%)
Not working/not in school	4 (10.0%)	1 (5.0%)	5 (8.3%)
Other	6 (15.0%)	1 (5.0%)	7 (11.7%)
Religion			
Christian	37 (92.5%)	17 (85.0%)	54 (90.0%)
Muslim	3 (7.5%)	3 (15.0%)	6 (10.0%)
Ethnic Tribe			
Luo	35 (87.5%)	16 (80.0%)	51 (85.0%)
Luhya	3 (7.5%)	1 (5.0%)	4 (6.7%)
Digo	1 (2.5%)	1 (5.0%)	2 (3.3%)
Baganda	0 (0%)	1 (5.0%)	1 (1.7%)
Other	1 (2.5%)	1 (5.0%)	2 (3.3%)
Length of time as Peer Educator			
Less than 1 year	N/A	1 (5.0%)	N/A
Between 1 and 2 years	N/A	11 (55.0%)	N/A
Between 2 and 5 years	N/A	6 (30.0%)	N/A
More than five years	N/A	2 (10.0%)	N/A

## Data Availability

The data presented in this study are available on request from the corresponding author. The data are not publicly available due to the highly sensitive nature of the data and the complexities involved in protecting the anonymity of participants in qualitative data.
